# Back squat and deadlift fatiguing protocols elicit distinct countermovement jump profiles: phase-specific predictors and soreness responses

**DOI:** 10.1093/bmb/ldag013

**Published:** 2026-04-19

**Authors:** Marco Gervasi, Eugenio Formiglio, Johnny Padulo, Giacomo Belmonte, Antonino Patti, Eneko Fernández-Peña

**Affiliations:** Department of Biomolecular Sciences, University of Urbino Carlo Bo, 61029 Urbino, Italy; Department of Biomolecular Sciences, University of Urbino Carlo Bo, 61029 Urbino, Italy; Department of Biomedical Sciences for Health, Università degli Studi di Milano, 20133 Milan, Italy; Department of Psychology, Educational Science and Human Movement, University of Palermo, 90144 Palermo, Italy; Department of Psychology, Educational Science and Human Movement, University of Palermo, 90144 Palermo, Italy; Department of Physical Education and Sport, University of the Basque Country UPV/EHU, 01007 Vitoria-Gasteiz, Spain

**Keywords:** countermovement jump, resistance exercise, neuromuscular fatigue, rate of force development, recovery kinetics

## Abstract

**Introduction or background:**

Residual fatigue following resistance exercise can impair neuromuscular performance. The back squat (BSQ) and deadlift (DL) are key lower-limb exercises but may differ in their fatigue and recovery profiles. Identifying phase-specific markers during the countermovement jump (CMJ) may improve monitoring and guide recovery strategies.

**Sources of data:**

Fifty-four resistance-trained adults (≥1 year experience) were randomly assigned to BSQ, DL, or control (CON) groups. After one-repetition maximum (1RM) estimation, BSQ and DL completed a fatiguing protocol (3 × 12 repetitions at 70% 1RM). CMJ performance was assessed at baseline, immediately, 30 min, and 24 h post-exercise using a force platform. Phase-specific force–time variables and the modified reactive strength index (RSImod) were calculated; muscle soreness was recorded at 24 and 48 h.

**Areas of agreement:**

Both BSQ and DL caused significant decrements in CMJ-derived metrics, as indicated by a linear mixed-effects model, and greater soreness than control, according to a two-way mixed analysis of variance (ANOVA). BSQ produced larger and earlier impairments across force, displacement, and work variables, whereas DL showed smaller but more persistent reductions in braking rate of force development (RFD) and RSImod.

**Areas of controversy:**

Despite similar soreness, recovery patterns differed, indicating distinct neuromuscular demands and time courses between exercises.

**Growing points:**

Phase-specific CMJ metrics particularly braking and unloading RFD, eccentric/concentric work, and RSImod are sensitive indicators of residual fatigue.

**Areas timely for developing research:**

Future studies should integrate electromyographic and biochemical markers and extend observation beyond 48 h to describe full recovery trajectories.

## Introduction

Fatigue is a multifactorial and complex phenomenon with substantial physiological and biomechanical implications for athletes [1–6]. It leads to reductions in maximal strength and power, impaired dynamic postural control, and slower reaction times, all of which contribute to decreased sports performance [1,2,7] and an elevated risk of injury [1,8,9]. Understanding and quantifying fatigue is therefore a fundamental objective for sports scientists and coaches seeking to optimize training adaptations while minimizing injury risk.

The vertical jump test is recognized as a reliable and minimally skill-dependent method for assessing lower-limb strength and power [10]. Among its variants, the countermovement jump (CMJ) is the most widely adopted because of its ease of administration and its capacity to provide detailed insight into lower-limb coordination, strength, power, and speed [11–15]. Traditionally, CMJ performance is quantified by jump height or kinematic and kinetic indices such as net relative impulse, which together represent gross indicators of neuromuscular output [16,17]. Beyond being a performance measure, CMJ-derived metrics have been validated as sensitive markers of neuromuscular readiness and overall fatigue status in both training and competition settings [18,19].

Numerous studies have explored how fatigue affects CMJ outcomes under various resistance or sport-specific protocols [20–24]. For example, Raeder *et al.* [20] demonstrated significant reductions in CMJ height following five dynamic squat exercise protocols, persisting at all post-exercise intervals. Cooper *et al.* [21] observed immediate decreases in jump height and peak power following continuous squat jumps. Turkmen *et al.* [22] found that both lower-limb and trunk fatigue protocols significantly reduced CMJ height, while Skala and Zemková [23] reported CMJ decrements within 10 minutes after small-sided soccer games. Similarly, Pareja-Blanco *et al.* [24] identified decreases in velocity and jump height lasting up to 48 h after resistance protocols inducing muscular failure. Collectively, these findings highlight that the CMJ is a sensitive and time-efficient tool for detecting acute and residual neuromuscular fatigue.

More recent analyses have shifted from global CMJ outcomes toward phase-specific predictors, examining the unloading, eccentric, and concentric phases of the movement [12,16]. This decomposition allows for a more nuanced understanding of how fatigue impairs specific mechanical functions, for instance, by reducing the yielding and braking rates of force development (RFD), or by delaying the transition between eccentric and concentric phases. These metrics capture distinct aspects of neuromuscular control that may not be evident through jump height alone [16]. Unfortunately, none of the studies discussed above assessed fatigue using phase-specific predictors, and consequently, little is known about how fatigue may influence these parameters. Identifying which CMJ phases are most sensitive to residual fatigue could therefore, in the future, provide practical insights for coaches in managing training loads, scheduling recovery, and designing interventions that enhance explosive performance while mitigating the risk of overuse injuries. At present, such translation to practice remains hypothetical, as specific fatigue thresholds for phase-specific predictors have yet to be established. Once defined, these thresholds could serve as valuable markers of athlete readiness, enabling more precise and individualized load management.

Resistance training protocols can elicit markedly different neuromuscular responses depending on the interaction between load intensity and training volume [25]. Protocols employing high intensities (≥85%–90% 1RM) and low repetitions (1–5 reps) are commonly used to induce post-activation potentiation or post-activation performance enhancement, aiming to transiently improve subsequent explosive performance [26,27]. However, these potentiation effects are often inconsistent and highly dependent on training background, rest intervals, and fiber-type composition [26,27].

In contrast, high-volume protocols performed at moderate intensities (~65%–75% of 1RM), commonly used in hypertrophy-focused resistance training, are known to induce significant acute neuromuscular fatigue that tends to outweigh any potentiating effects [24,25,28]. This fatigue arises from both peripheral and central mechanisms. On the peripheral side, repeated contractions with short inter-repetition recovery lead to metabolic stress (accumulation of inorganic phosphate, hydrogen ions, and lactate) and impairments in excitation–contraction coupling, reducing calcium ion release and cross-bridge cycling efficiency [29,30]. Moreover, prolonged time under tension accelerates glycogen depletion, alters intramuscular ionic gradients, and increases reliance on type II fibers, amplifying fatigue in high-threshold motor units [31,32].

At the neural level, sustained effort across multiple repetitions recruits progressively higher-threshold motor units according to the Henneman size principle [33]. As low-threshold (type I) fibers fatigue, the nervous system compensates by recruiting additional type IIa and IIx fibers to maintain force output [34]. This sequential recruitment under metabolic stress leads to near-complete activation of the motor pool, resulting in global neuromuscular fatigue that is both peripheral (muscle-level) and central (reduced voluntary drive and motor unit firing rates) [31]. Such protocols therefore represent a robust model to elicit multifactorial fatigue, mimicking the demands of high-effort hypertrophy or mixed-strength training commonly used in athletic contexts.

Several studies have empirically confirmed that moderate-intensity, high-volume resistance sessions (6–12 repetitions at ~ 70% 1RM) induce larger decrements in jump height, maximal voluntary contraction, and RFD compared to heavier, low-repetition sets [24,25,35]. Pareja-Blanco *et al.* [24] and Davies *et al.* [36] demonstrated that when repetitions approach failure, velocity loss becomes a key predictor of neuromuscular fatigue, often requiring > 24 h for full recovery. From a biomechanical perspective, such fatigue-related mechanisms are expected to primarily affect CMJ phase-specific variables, particularly reducing eccentric Braking RFD, impairing the efficiency of the stretch–shortening cycle, and lowering concentric peak force and impulse during the propulsive phase, ultimately resulting in decreased jump height.

The back squat (BSQ) and deadlift (DL) are foundational resistance exercises that place substantial demands on the neuromuscular system. While both exercises induce significant acute fatigue when performed with high loads and volume, comparative evidence suggests that their neuromuscular fatigue profiles may differ. Previous studies comparing squat and DL protocols have reported similar reductions in maximal voluntary force and voluntary activation, with a greater peripheral fatigue response following squat exercise, likely due to the higher quadriceps involvement, while no clear differences in acute endocrine responses have been observed between exercises [37]. More broadly, systematic evidence indicates that increasing intensity and fatigue in compound lifts consistently leads to reductions in velocity and power output, with exercise-specific kinetic and kinematic adaptations, although deadlift-specific fatigue responses remain underrepresented in the literature [38].

Therefore, the aim of this study was to investigate the acute and short term effects of moderate-intensity, high-volume BSQ and DL exercises on CMJ performance and phase-specific metrics. We hypothesized that BSQ would induce a larger immediate decrement in the primary outcome (jump height) compared with DL, whereas DL would show a more persistent impairment in secondary indicators of rapid force production and reactivity (e.g. Braking RFD and modified reactive strength index [RSImod]). We also expected greater DOMS following both resistance protocols compared with control.

## Materials and methods

### Participants

Fifty-six resistance-trained participants (11 females and 45 males; age: 21.4 ± 2 years; height: 175.4 ± 7.5 cm; body mass: 73.5 ± 11.3 kg; body mass index: 23.8 ± 2.7 kg/m^2^) volunteered to participate in the study. Inclusion criteria were being healthy at the time of the study, aged between 18 and 30 years, and having at least one year of structured resistance-training experience. All participants reported engaging in resistance training 3–5 sessions per week over the previous 12 months, with an average session duration of ~1.5 hours. All participants regularly performed both the DL and the BSQ as part of their standard training routines.

Strength assessments conducted prior to the experimental protocol indicated a moderately to well-trained profile, with mean estimated 1RM values of 169.3 ± 40.6 kg (2.17 ± 0.5 × body mass) for the DL in the DL group, and 121.0 ± 28.0 kg (1.71 ± 0.2 × body mass) for the BSQ in the BSQ group. These values are consistent with established resistance-training classification frameworks that categorize individuals with this combination of training experience, technical proficiency, and relative strength as intermediate/advanced [39].

Exclusion criteria included smoking, consuming more than three alcoholic drinks per day, and having acute or chronic diseases or treatment with drugs affecting muscle recovery and musculoskeletal performance. After being informed of the aims, procedures, and risks of the exercise protocol and following a medical health screening, each subject signed a written informed consent form. The protocol was approved by the Ethics Committee of the University of Palermo, Italy (n. 311/2005-Prot.62141; April 24, 2025) and was conducted in accordance with the Declaration of Helsinki (Fortaleza revision) for research with human volunteers [40]. All participants were assessed for height and weight, and the Body Mass Index was calculated. Before commencing the experimental phase, participants took part in a familiarization session with the procedures.

### Study design

The initial allocation plan was to include 12 participants in the control group and 22 participants in each experimental group (BSQ and DL) to prioritize statistical power for the main comparisons between the two resistance fatiguing exercise protocols. Randomization was stratified by sex, as there were 11 female participants: 1 was allocated to the control group, 5 to the BSQ group, and 5 to the DL group. Participants were randomly assigned within these strata using a computer-generated random sequence. Unfortunately, 2 female participants originally assigned to the DL group withdrew after the first session for personal reasons, resulting in the final sample distribution: control (CON, 11 males and 1 female), BSQ (17 males and 5 females), and DL (17 males and 3 females).

All participants then completed a familiarization protocol consisting of five sets of three repetitions of CMJs. These sessions were supervised by a qualified researcher who provided instructional guidance and corrective feedback to ensure proper technique.

One week later, during the second session, participants performed an incremental load test to estimate their one-repetition maximum (1RM) for the BSQ using a Smith machine and for the DL using free weight.

The third session, conducted 48 h later, began with a standardized warm-up comprising 5 minutes of fast-paced treadmill walking (5–6.5 km/h) followed by 5 minutes of mobilization exercises targeting both upper and lower limbs. Participants then performed three CMJs to establish baseline performance (T0). Following this, the BSQ and DL groups completed specific preparatory sets to ensure readiness and correct execution before engaging in a fatiguing resistance exercise protocol consisting of three sets of 12 repetitions of their assigned exercise. The time required to complete the fatiguing protocol was ~15 minutes (including warm-up) for each participant; therefore, the CMJ test at T1 was conducted ~15 minutes after T0 for BSQ and DL groups. To maintain consistency in timing across groups, the CON group rested for 15 minutes before performing the CMJ test at T1. All participants repeated the CMJ test again 30 minutes after T1 (T30 min).

The fourth session, conducted 24 h after the fatiguing protocol (T24h), included an assessment of post-exercise muscle soreness and a repetition of the CMJ test initially performed at baseline (T0). Due to scheduling conflicts, CMJ data at T24h is missing for 5 participants in the BSQ group and 3 participants in the DL group. Additionally, post-exercise muscle soreness was assessed again 48 h after the fatiguing protocol to capture muscle soreness progression.

### Instruments

To assess the displacement velocity of the load, a validated linear position transducer consisting of an optical encoder for measuring angular movements and a coil for linearization was used, allowing precise measurement of linear movements (Vitruve, Speed4lifts, Móstoles, Madrid, Spain) [41–43]. To measure the vertical ground reaction force (GRF) and calculate the phase-specific predictors of CMJs, a force platform (MuscleLab™ system, type PFMA 3010e) sampling at 100 Hz was used [44]. A Smith machine with no counter-weight mechanism (Multipower Technogym, Cesena, Italy) was used for BSQ resistance exercise sessions.

### Procedures

#### 1RM estimation

Individual 1RM estimations were determined using a progressive loading test in both the BSQ and DL exercises. The BSQexercise was performed using a Smith machine as a deliberate design choice to enhance participant safety and ensure standardized movement execution, particularly during high-volume squatting, which carries a higher risk of technical errors and injury. Participants began in an upright standing position with knees and hips fully extended. From this position, they descended in a controlled manner until the upper thighs were positioned below the horizontal plane, indicating a full-depth squat. Immediately upon reaching the lowest point, participants reversed the movement and ascended to the starting position, executing the concentric phase with maximal intentional velocity. The DL was performed with a standard Olympic barbell. Participants initiated the movement from a standing position with the barbell resting on the floor, feet shoulder-width apart, and hands gripping the bar just outside the knees. With a neutral spine and engaged core, they lifted the barbell by extending the hips and knees simultaneously until reaching full upright posture. The barbell was then lowered in a controlled manner back to the floor, completing one repetition. Each lift was executed with maximal intentional velocity during the concentric phase.

To determine 1RM, each participant began with a self-selected load they could confidently lift with proper technique. The load was increased gradually across attempts until the predetermined velocity threshold was reached. Each attempt was separated by a standardized 3-minute rest interval to minimize fatigue effects. Participants typically performed 4–6 attempts, depending on their initial load selection and velocity profile.

Barbell velocity was recorded on every repetition, and the test was terminated when one of the following criteria was met: (i) the mean concentric velocity of the propulsive phase fell to ≤ 0.05 m/s [45]; (ii) the participant failed to complete the repetition with correct technique; or (iii) the participant reported excessive discomfort or declined to attempt a higher load. Finally, the linear regression equation was determined using literature-based minimum velocity thresholds of 0.30 m/s for the BSQ and 0.15 m/s for the DL [46], while repetitions performed at a mean velocity above 1.0 m/s were not considered. The individual load corresponding to 70% of 1RM was determined and used for the 3 x 12 fatiguing protocol [24].

#### CMJ test

Participants performed three CMJs, each separated by a 30-second rest interval. All jumps were conducted under the supervision of two physical exercise experts, who instructed participants to begin the concentric phase from a knee angle of ~90° and to jump as high as possible while keeping their hands on their hips [23,47]. If a technical error occurred during the execution of a jump (such as an incorrect squat depth, an additional preparatory movement, or a clear lack of maximal effort) the participant was asked to repeat the attempt. To ensure measurement reliability, the mean value of the three valid jumps was used for subsequent analysis.

#### Fatiguing resistance exercise protocols (FREP)

The warm-up preceding the BSQ and DL fatiguing protocols consisted of three sets of six repetitions of full squats or DLs performed with progressively increasing loads of 40%, 50%, and 60% of 1RM, with a one-minute rest interval between sets. After a two-minute rest period, participants performed the fatiguing resistance exercise protocols, which comprised three sets of 12 repetitions (3 x 12) of full squats or DLs at 70% of 1RM. Each set was separated by a two-minute rest interval. During both the warm-up and fatiguing protocols, participants were instructed to perform all repetitions at maximal intended velocity.

#### Phase-specific predictors of CMJ and delayed-onset muscle soreness (DOMS)

To quantify the neuromuscular fatigue induced by the 3 x 12 FREP, changes in CMJ phase-specific predictors were assessed before and after exercise. The variables analyzed related to the biomechanics of the CMJ [12,16] were divided into specific categories.

Position and displacement-related variables were: flight height (in m), defined as the total vertical displacement of the centre of mass (CoM) from the point of take-off to the peak of the jump and calculated using impulse-momentum method [48]; jump height (in m), defined as the total vertical displacement of the CoM from the standing position to the peak of the jump and calculated by double integration from GRF; minimum position of CoM (PosMin, in m), defined as the lowest position of CoM reached during the countermovement of the CMJ.

Force-related variables were: RFD during the braking phase (Braking RFD, in N/kg/s); RFD during the yielding phase (Yielding RFD, in N/kg/s); RFD during the unloading phase (Unloading RFD, in N/kg/s); force at minimum position (F@PosMin, in N), defined as the vertical GRF at PosMin; minimum force (Fmin, in N), defined as the minimum GRF at the end of the unloading phase; average concentric force (FconAvg, in N), defined as the average force generated during the concentric phase.

Time or duration-related variables were: Unloading duration (in s), defined as the time interval from the moment the GRF decreased by more than 2.5% of the calculated body mass value to the point at which the minimum GRF was reached during the jump; Yielding duration (in s), defined as the time interval from Fmin to the peak negative CoM velocity; Braking duration (in s), defined as the time interval from the peak negative CoM velocity until PosMin; Eccentric duration (in s), defined as the sum of Yielding and Braking durations; Concentric duration (in s), defined as the time interval from PosMin to take-off; Total Jump Time (in s), defined as the sum of Unloading, Yielding, Braking and Concentric durations.

Work and energy-related variables were: total mechanical work during the concentric phase (Wcon, in J); total mechanical work during the eccentric phase (Wecc, in J).

A composite variable was also measured to quantify the neuromuscular performance index: RSImod (in m/s), defined as flight height divided by Total Jump Time [49].

Finally, delayed onset muscle soreness (DOMS) was assessed 24 and 48 h after completing the FREP protocol to evaluate residual fatigue. Participants verbally rated their global lower-limb soreness (not specific to individual muscle groups) using the Numeric Pain Rating Scale (NPRS), which consists of 11 points ranging from 0 (no pain) to 10 (extreme pain) [50–56]. This method was chosen for its efficiency, requiring less than one minute to administer, and for its simplicity and reliability in evaluating muscle soreness intensity [50,53,57,58]. DOMS at 24 h (DOMS24h) was measured in person during the fourth session, immediately before the warm-up, while DOMS at 48 h (DOMS48h) was assessed through a follow-up phone call. Both ratings were collected by the same researcher, who administered the scale consistently across time points using identical wording and anchoring.

#### Statistical analysis

All statistical analyses were conducted using GraphPad Prism 8.0 (GraphPad Software, San Diego, CA, USA). Given the repeated-measures design and the presence of some missing data, a linear mixed-effects model (LMM) with restricted maximum likelihood estimation was applied to examine the fixed effects of time (T0, T1, T30 min, T24h), group (CON, BSQ, DL), and their interaction (time × group) for each biomechanical variable derived from the CMJ. LMM estimations were performed under the assumption that missing data were missing at random (MAR). To control for interindividual baseline variability, all outcome measures were normalized by computing percentage changes from baseline (T0), which was set to 0% for each participant and calculated as %Δ = ((Tx − T0)/T0) × 100, where Tx denotes the value at T1, T30 min, or T24h. This normalization allowed for a clearer interpretation of fatigue-related changes across groups and time points. Multiple comparisons were adjusted using Dunnett’s post hoc test for CMJ-derived variables, and statistical significance was set at *P* < 0.05. Data are reported as mean ± standard deviation (SD) unless stated otherwise. Interaction effects were interpreted based on the magnitude and direction of percentage changes relative to the CON group.

In addition to the main statistical tests, standardized effect sizes were computed to quantify the magnitude of within-group changes over time. For CMJ variables, paired Cohen’s *d* values were calculated using absolute scores for each key contrast (T0-T1, T0-T30 min, T0-T24h), defined as the mean of the paired differences divided by their standard deviation. Ninety-five percent confidence intervals (95% CI) for *d* were obtained using the standard error formula for paired designs. Direct comparisons between the DL and BSQ groups were not pre-specified as primary hypotheses and were therefore treated as merely descriptive.

Muscle soreness was assessed using the NPRS (0–10) at 24 h and 48 h post-exercise and analyzed using a two-way mixed analysis of variance (ANOVA), with Time (T24h, T48h) as the within-subjects factor and Condition (CON, DL, BSQ) as the between-subjects factor. Partial η^2^ values were computed for the main effects (Time, Condition) and their interaction within the two-way mixed ANOVA. When significant main effects or interactions were detected, Tukey’s HSD post hoc tests were applied for pairwise comparisons. Statistical significance was set at *P* < 0.05, and results were reported as mean differences and percentage variations relative to the control condition.

## Results

**Figure 1:**
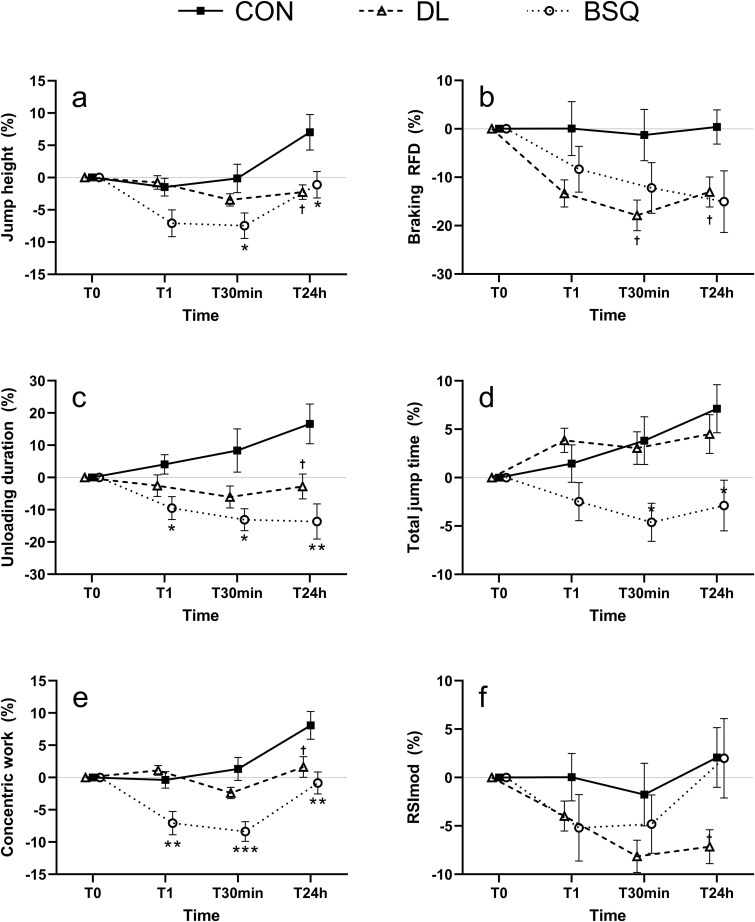
Representative percentage changes in selected CMJ variables relative to baseline (T0) for the three conditions: Control (CON), deadlift (DL), and back squat (BSQ). Baseline (T0) is set as 0%. Values are presented as mean ± standard error. a) total vertical displacement of the Centre of mass from the standing position to the peak of the jump (jump height); b) RFD in the braking subphase (braking RFD); c) duration of the unloading phase (unloading duration); d) total duration of the jump, from the start of the unloading phase to the end of the concentric phase (Total jump time); e) work performed during the concentric phase (concentric work); f) flight height divided by Total jump time (RSImod). ^*^ statistically significant between BSQ and CON (*P* < 0.05); ^**^ statistically significant between BSQ and CON (*P* < 0.01); ^***^ statistically significant between BSQ and CON (*P* < 0.001); † statistically significant between DL and CON (*P* < 0.05).

The outcomes of the linear mixed-effects analysis are presented in Tables 1–4. Representative changes in selected CMJ variables are depicted in Fig. 1. Each table cell reports the within-condition percentage change from baseline (T0) followed by the between-condition difference relative to the control (Δ vs CON). All *P*-values refer exclusively to comparisons with the CON condition.

**Table 1:** Force-related variables: Force-related variables derived from countermovement jump analysis: Rate of force development in the braking subphase (braking RFD); rate of force development in the yielding subphase (yielding RFD); rate of force development in the unloading phase (unloading RFD); force expressed at the lowest position of the Centre of mass (force at PosMin); lowest force value recorded before the lift (Minimum force); average force expressed during the concentric phase (average concentric force). For each time point, the first value represents the within-condition percentage change from baseline (T0), whereas the second value (Δ vs CON) represents the difference between the control and experimental conditions at the same time point (Δ vs CON = CON − experimental condition).

Variable	Condition	T1 (Δ vs CON; p)	T30 min (Δ vs CON; p)	T24h (Δ vs CON; p)	Significance
Braking RFD	Deadlift	−13.34% (+13.37%; 0.08)	−17.89% (+16.61%; **0.03**)	−13.04% (+13.41%; **0.02**)	At T30 min and T24h
	Back squat	−8.33% (+8.37%; 0.40)	−12.22% (+10.94%; 0.25)	−15.06% (+15.43%; 0.08)	n.s.
Yielding RFD	Deadlift	−4.59% (+2.08%; 0.87)	−3.23% (+2.04%; 0.92)	−4.69% (−4.15%; 0.73)	n.s.
	Back squat	+4.12% (−6.62%; 0.58)	+26.60% (−31.86%; **0.01**)	+29.24% (−38.09%; **0.007**)	At T30 min and T24h
Unloading RFD	Deadlift	+7.87% (−7.58%; 0.73)	+18.79% (−0.97%; > 0.99)	+11.44% (+0.78%; > 0.99)	n.s.
	Back squat	+11.47% (−11.18%; 0.53)	−3.58% (+21.40%; 0.14)	−21.78% (+33.99%; **0.01**)	At T24h
Force at PosMin (F@PosMin)	Deadlift	−4.50% (+3.95%; 0.13)	−6.94% (+5.99%; **0.03**)	−3.00% (+3.28%; 0.10)	At T30 min
	Back squat	−5.21% (+4.65%; 0.14)	−6.29% (+5.34%; 0.08)	−4.70% (+4.99%; 0.11)	n.s.
Minimum force (Fmin)	Deadlift	+4.09% (+2.64%; 0.98)	+12.70% (+9.67%; 0.75)	+10.42% (+18.19%; 0.46)	n.s.
	Back squat	+2.25% (+4.49%; 0.93)	−14.66% (+37.03%; **0.005**)	−29.32% (+57.93%; **0.005**)	At T30 min and T24h
Average concentric force (FconAvg)	Deadlift	−2.58% (+2.28%; 0.07)	−3.15% (+1.73%; 0.27)	−2.69% (+1.33%; 0.53)	n.s.
	Back squat	−3.12% (+2.83%; 0.13)	−3.01% (+1.59%; 0.49)	−1.07% (−0.29%; 0.98)	n.s.

*P*-values correspond exclusively to the comparison with the control condition. Statistical analysis was performed using linear mixed-effects models (LMM) including condition, time, and their interaction as fixed effects, and participant as a random intercept. Bold values indicate statistically significant differences (*P* < 0.05); n.s. = not significant; trend-level = 0.05 ≤ *P* < 0.10.

**Table 2:** Time- or duration-related variables: Temporal and duration-related parameters characterizing the countermovement jump phases: Total duration of the jump, from the start of the unloading phase to the end of the concentric phase (Total jump time); duration of the unloading phase (unloading duration); duration of the yielding subphase (eccentric yielding duration); duration of the braking subphase (eccentric braking duration); total duration of the eccentric phase, sum of the durations of the yielding and braking subphases (total eccentric duration); duration of the concentric phase (concentric duration). Each cell reports the within-condition percentage change from baseline (T0), followed by the between-condition difference relative to control (Δ vs CON).

Variable	Condition	T1 (Δ vs CON; *P*)	T30 min (Δ vs CON; *P*)	T24h (Δ vs CON; *P*)	Significance
Total jump time	Deadlift	+3.85% (−2.39%; 0.47)	+3.06% (+0.75%; 0.95)	+4.51% (+2.62%; 0.62)	n.s.
	Back squat	−2.48% (+3.95%; 0.26)	−4.61% (+8.42%; **0.02**)	−2.88% (+10.01%; **0.02**)	At T30 min and T24h
Unloading duration	Deadlift	−2.52% (+6.58%; 0.25)	−6.03% (+14.39%; 0.12)	−2.75% (+19.36%; **0.03**)	At T24h
	Back squat	−9.47% (+13.53%; **0.01**)	−13.10% (+21.46%; **0.02**)	−13.61% (+30.21%; **0.002**)	At all time points
Eccentric Yielding duration	Deadlift	+5.48% (−2.33%; 0.81)	+5.47% (−1.77%; 0.94)	+6.52% (+4.12%; 0.78)	n.s.
	Back squat	−0.29% (+3.45%; 0.65)	−8.35% (+15.58%; **0.05**)	−8.51% (+19.15%; **0.04**)	At T30 min and T24h
Eccentric braking duration	Deadlift	+8.82% (−7.45%; ~ 0.05)	+10.09% (−8.28%; **0.05**)	+9.74% (−7.81%; 0.15)	Trend-level at T1–T30 min
	Back squat	+3.52% (−2.16%; 0.80)	+5.97% (−4.15%; 0.48)	+12.09% (−10.15%; 0.13)	n.s.
Total eccentric duration	Deadlift	+6.92% (−5.09%; 0.18)	+7.55% (−4.28%; 0.36)	+7.75% (−3.19%; 0.66)	n.s.
	Back squat	+1.09% (+0.73%; 0.96)	−1.07% (+4.34%; 0.36)	+1.30% (+3.26%; 0.60)	n.s.
Concentric duration	Deadlift	+5.09% (−4.89%; **0.05**)	+4.37% (−1.30%; 0.82)	+6.97% (−0.43%; 0.99)	Trend-level at T1
	Back squat	+0.01% (+0.19%; > 0.99)	−1.48% (+4.55%; 0.22)	+2.26% (+4.28%; 0.36)	n.s.

*P*-values are derived from linear mixed-effects models (LMM) assessing the effect of condition, time, and their interaction, with subject included as a random effect. Bold values indicate statistically significant differences (*P* < 0.05); n.s. = not significant; trend-level = 0.05 ≤ *P* < 0.10.

**Table 3:** Position- and displacement-related variables: Position- and displacement-related variables obtained from countermovement jump analysis: Lowest position reached by the Centre of mass during the jump (PosMin); total vertical displacement of the Centre of mass from the point of take-off to the peak of the jump (flight height); total vertical displacement of the Centre of mass from the standing position to the peak of the jump (jump height). Each cell reports the within-condition percentage change from baseline (T0), followed by the between-condition difference relative to control (Δ vs CON).

Variable	Condition	T1 (Δ vs CON; *P*)	T30 min (Δ vs CON; *P*)	T24h (Δ vs CON; *P*)	Significance
PosMin	Deadlift	+4.23% (−2.25%; 0.70)	−0.51% (+5.28%; 0.28)	+6.00% (+6.54%; 0.34)	n.s.
	Back squat	−6.87% (+8.85%; **0.03**)	−9.32% (+14.10%; **0.005**)	−1.05% (+13.59%; **0.02**)	At all time points
Flight Height	Deadlift	−0.64% (+1.56%; 0.49)	−5.86% (+7.12%; **0.01**)	−3.42% (+12.16%; **0.002**)	At T30 min and T24h
	Back squat	−8.40% (+9.32%; **0.005**)	−9.79% (+11.04%; **0.002**)	−1.66% (+10.40%; **0.02**)	At all time points
Jump Height	Deadlift	−0.75% (−0.74%; 0.87)	−3.47% (+3.33%; 0.29)	−2.26% (+9.27%; **0.01**)	At T24h
	Back squat	−7.08% (+5.59%; 0.06)	−7.45% (+7.32%; **0.04**)	−1.12% (+8.13%; **0.05**)	At T30 min and T24h

*P*-values are from linear mixed-effects models (LMM). Bold values indicate statistically significant differences (*P* < 0.05).

**Table 4:** Work, energy, and composite variables: Work-, energy-, and neuromuscular performance-related variables: Work performed during the concentric phase (concentric work); work performed during the eccentric phase (eccentric work); flight height divided by Total jump time or modified reactive strength index (RSImod). Each cell reports the within-condition percentage change from baseline (T0), followed by the between-condition difference relative to control (Δ vs CON).

Variable	Condition	T1 (Δ vs CTRL; *P*)	T30 min (Δ vs CTRL; *P*)	T24h (Δ vs CTRL; *P*)	Significance
Concentric Work	Deadlift	+1.11% (−1.47%; 0.51)	−2.38% (+3.72%; 0.13)	+1.63% (+6.45%; **0.05**)	Trend-level at T24h
	Back squat	−7.07% (+6.71%; **0.009**)	−8.36% (+9.70%; **< 0.001**)	−0.85% (+8.92%; **0.007**)	At all time points
Eccentric Work	Deadlift	+4.61% (−3.38%; 0.49)	−0.04% (+4.51%; 0.41)	+7.05% (+4.86%; 0.56)	n.s.
	Back squat	−6.64% (+7.87%; 0.08)	−9.42% (+13.89%; **0.008**)	−0.37% (+12.28%; **0.04**)	At T30 min and T24h
RSImod	Deadlift	−3.98% (+4.02%; 0.29)	−8.14% (+6.37%; 0.16)	−7.14% (+9.22%; **0.03**)	At T24h
	Back squat	−5.19% (+5.24%; 0.35)	−4.80% (+3.04%; 0.70)	+2.00% (+0.08%; > 0.99)	n.s.

*P*-values were obtained from linear mixed-effects models (LMM). Bold values indicate statistically significant differences (*P* < 0.05).

To facilitate full contextualization of the percentage-change results, absolute mean ± SD values for all CMJ variables at each time point and for all groups (corresponding to the variables reported in Tables 1–4) are provided in the Supplementary Material (Table S1). The same table also includes paired Cohen’s *d* effect sizes with corresponding 95% confidence intervals. Overall, the computed effect sizes ranged from trivial to moderate depending on the variable and time contrast.

In the force-related variables (Table 1), the DL condition showed significant reductions in Braking RFD at T30 min (−17.9%; *P* = 0.03) and T24h (−13.0%; *P* = 0.02), whereas the BSQ condition exhibited no significant differences at any time point (all *P* > 0.05). Yielding RFD increased significantly in the BSQ condition at T30 min and T24h (*P* = 0.01 and 0.007), while Unloading RFD was reduced at T24h (*P* = 0.01). F@PosMin decreased significantly in the DL condition at T30 min (*P* = 0.03), and Minimum Force (Fmin) displayed the largest decrements in the BSQ condition at T30 min and T24h (both *P* = 0.005). No significant effects were detected for Average Concentric Force.

Among the time or duration-related variables (Table 2), Total Jump Time was significantly longer in the BSQ condition at T30 min and T24h (*P* = 0.02 and 0.02). Unloading duration was significantly shorter in the BSQ condition across all time points (T1: *P* = 0.01; T30 min: *P* = 0.02; T24h: *P* = 0.002), whereas the DL condition showed a reduction only at T24h (*P* = 0.03). Eccentric Yielding duration decreased significantly in the BSQ condition at T30 min and T24h (*P* = 0.05 and 0.04). Eccentric Braking duration and Concentric duration in the DL condition showed trend-level increases at early time points (*P* ≈ 0.05), while no other temporal parameters changed significantly.

Regarding the position- and displacement-related variables (Table 3), the BSQ condition showed consistent decreases in PosMin across all time points (T1: *P* = 0.03; T30 min: *P* = 0.005; T24h: *P* = 0.02). Flight Height decreased significantly at T1, T30 min and T24h in BSQ conditions while only at T30 min and T24h in DL, with the largest reduction occurring in the BSQ condition (T30 min: *P* = 0.002). Jump Height declined significantly at T24h in the DL condition (*P* = 0.01) and at T30 min and T24h following the BSQ protocol (*P* = 0.04 and 0.05).

In the work-, energy-, and composite-related variables (Table 4), Concentric Work decreased significantly after the BSQ condition at all time points (T1: *P* = 0.009; T30 min: *P* < 0.001; T24h: *P* = 0.007), whereas a trend-level reduction was observed in the DL condition at T24h (*P* = 0.05). Eccentric Work was significantly lower in the BSQ condition at T30 min (*P* = 0.008) and T24h (*P* = 0.04). Finally, the composite neuromuscular index RSImod declined significantly at T24h in the DL condition (*P* = 0.03), with no corresponding change following the BSQ protocol.

Overall, the BSQ protocol elicited broader and more persistent impairments across force, temporal, and displacement parameters compared with the DL condition, indicating a greater neuromuscular load and a slower recovery profile following BSQ exercise.

**Figure 2:**
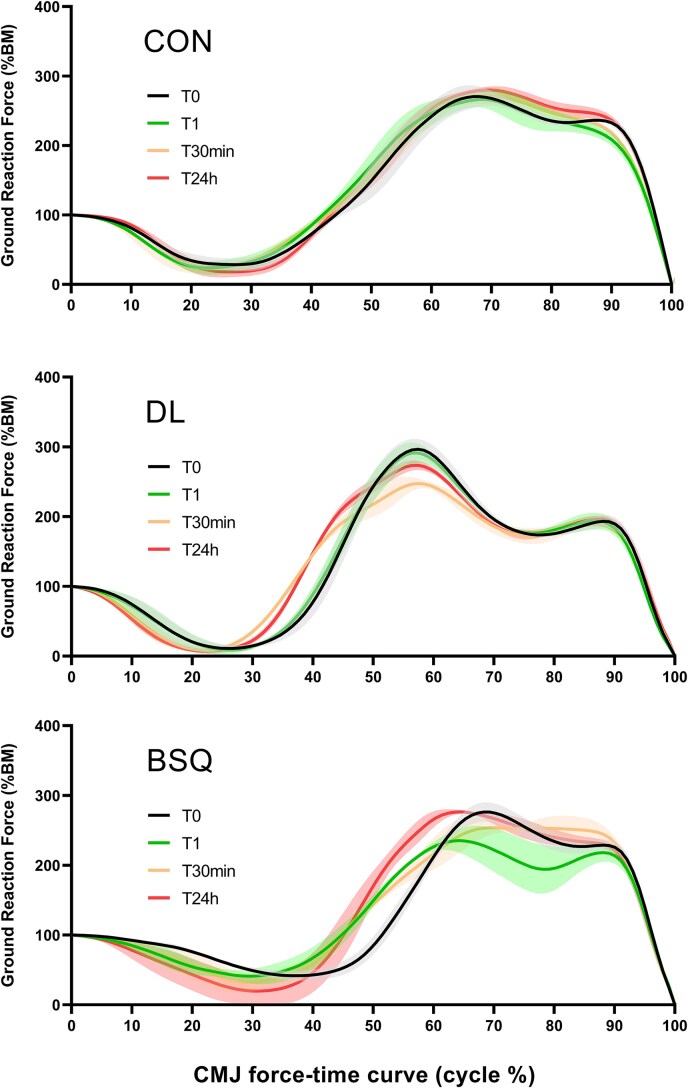
Ensemble-averaged CMJ force–time profiles at four time points: T0 (black), T1 (green), T30 min (orange), and T24h (red) for one participant from each condition: Control (CON), deadlift (DL), and back squat (BSQ). For each participant, condition, and time point, the three CMJs were normalized to 100% of body mass (%BM) and then to 100% of total jump duration, before averaging. Shaded areas indicate ±1 standard deviation.

Figure 2 illustrates representative ensemble-averaged CMJ force–time profiles at four time points (T0, T1, T30 min, and T24h) for one participant from each condition (CON, DL, and BSQ). Profiles were normalized to body mass (%BM) for comparability. These examples are shown for illustrative purposes and might not be representative of all participants.

### Delayed onset muscle soreness

**Figure 3:**
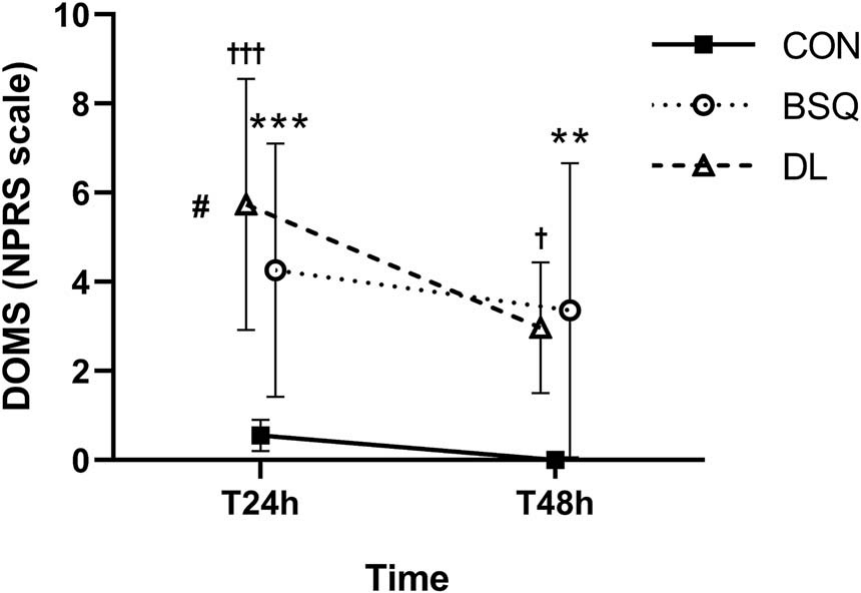
Delayed onset muscle soreness (DOMS) for control (CON), deadlift (DL), and back squat (BSQ) conditions at T24h and T48h time points, measured with the numerical pain rating scale (NPRS) from 0 to 10. Values are presented as mean ± standard error. ^**^ statistically significant between BSQ and CON (*P* < 0.01); ^***^ statistically significant between BSQ and CON (*P* < 0.001); † statistically significant between DL and CON (*P* < 0.05); ††† statistically significant between DL and CON (*P* < 0.001); # statistically significant between DL at T24h and DL at T48h (*P* < 0.05).

The two-way repeated-measures ANOVA for NPRS scores (Fig. 3) revealed significant main effects of time (*F* = 8.321, *P* = 0.005, partial *η*^2^ = 0.352) and condition (*F* = 24.025, *P* < 0.001, partial *η*^2^ = 0.379), with no significant time × condition interaction (*F* = 2.049, *P* = 0.135, partial *η*^2^ = 0.233). Post hoc comparisons (Tukey’s HSD) indicated that perceived muscle soreness was significantly higher at T24h than at T48h (mean difference = 1.41 ± 0.49, *P* = 0.005), reflecting an overall decrease in soreness over time across conditions. Across conditions, both BSQ (mean difference vs Control = 3.53 ± 0.62, *P* < 0.001) and DL (mean difference vs Control = 4.08 ± 0.62, *P* < 0.001) elicited significantly greater DOMS compared with the Control condition, while no significant difference was detected between BSQ and DL (*P* = 0.592). The time × condition post hoc tests confirmed that at T24h both resistance exercise conditions induced substantially higher soreness than the Control group (BSQ: *P* < 0.001; DL: *P* = 0.041). By T48h, soreness declined in both conditions but remained significantly greater than Control (BSQ: *P* < 0.001; DL: *P* = 0.013). The graphical pattern (Fig. 3) illustrates this temporal trend, with peak soreness at T24h and partial recovery by T48h, particularly pronounced in the DL condition, which showed a steeper decline.

## Discussion

This study examined the acute (T1, T30 min) and short-term (T24h) effects of moderate-intensity, high-volume BSQ and DL fatiguing protocols on phase-specific CMJ variables, alongside DOMS at 24 and 48 h. Confirming our hypotheses, BSQs induced a larger acute decrement in jump height, whereas DLs produced smaller acute reductions but more persistent alterations in phase-specific indicators of rapid force production and reactivity.

### Immediate acute effects (T1) on jump performance and kinetics

Immediately after the exercise, marked differences emerged between DL and BSQ conditions in vertical jump parameters. The BSQ induced a significant reduction in jump height and flight height immediately post-exercise, whereas DL did not show any appreciable decrease at the same time point. This finding is consistent with previous studies comparing the acute effects of the two exercises, which have reported that squats markedly reduce CMJ height, whereas heavy DLs show either no immediate decline or a more modest one [35,59]. However, these studies employed variations of the lifts (e.g. free-weight or trap-bar DLs) that are biomechanically similar but not identical to the protocols used in the present experiment; therefore, direct comparison should be interpreted with caution.

Biomechanically, this difference can be explained by distinct muscle emphasis: the BSQ is a knee-dominant movement with high activation of quadriceps and knee extensors, while the DL is hip-dominant, involving mainly the posterior chain (gluteus, hamstrings, and spinal erectors) and relatively less quadriceps activation [60]. Consequently, a heavy squat directly fatigues the main jumping muscles (quadriceps and gluteus) more than a DL, resulting in a greater immediate loss of height and reactivity indices.

Beyond the drop in performance (jump height, flight height, and RSImod), the force–time curve analysis revealed immediate changes in movement strategy (Fig. 2). In the BSQ condition, participants performed a shallower and faster countermovement at T1: the minimum position was reduced, and the unloading phase was shorter. Such adaptation, widely reported under fatigue, reduces eccentric work and elastic energy storage, thereby decreasing concentric impulse and power output [61]. After DL, no significant reductions in countermovement depth or eccentric phase duration were observed, in line with its lower immediate interference on jump mechanics.

In terms of rate of force development, Braking RFD tended to decrease in both groups at T1 but more prominently in the DL (Fig. 1b), while in the BSQ it was slightly attenuated, possibly because reduced depth and descent speed limited the braking demand. Yielding RFD exhibited small, non-significant changes (a slight drop after BSQ, a minor increase after DL), which must be interpreted cautiously.

### Short-term recovery (30 min)

Thirty minutes post-exercise, residual neuromuscular fatigue persisted in both groups, with distinct patterns. In the BSQ group, jump height remained below baseline (Fig. 1a), and both eccentric and concentric work outputs were still depressed (Fig. 1e). The Unloading duration was reduced (Fig. 1c), and Yielding RFD paradoxically increased, possibly reflecting a compensatory strategy to ‘drop’ faster at the beginning of the countermovement and exploit stored elastic energy despite fatigue [61]. In contrast, in the DL condition, some alterations became more evident at 30 min compared to immediately after: declines in flight height and Braking RFD were noted, accompanied by slight prolongation of eccentric durations, suggesting a delayed manifestation of fatigue.

### Recovery and residual effects at 24 h

At T24h, most CMJ performance parameters (particularly jump height) had returned to values close to baseline in both exercise groups. However, when compared with the control condition, subtle residual differences were still present. Control participants exhibited small performance improvements at T24h (Fig. 1a), likely reflecting familiarity with the testing procedures or simply greater freshness, whereas participants in the BSQ and DL groups generally recovered only to their baseline levels, with no evidence of supercompensation.

Jump height and flight height represent direct indicators of neuromuscular performance during the CMJ. Substantial evidence indicates that both parameters exhibit an immediate decline following high-intensity resistance exercise, with recovery to baseline values complete or partial occurring within 24–48 h, depending on factors such as exercise modality, volume, and repetition scheme [24]. For instance, Kotikangas *et al*. (2025) [62] reported a significant CMJ height reduction immediately following 5 × 10 RM squats, with return to baseline within 24 h A similar pattern was observed in this study: the BSQ group exhibited a pronounced acute decrease in jump height, whereas the DL group showed a smaller immediate reduction (Fig. 1a). This distinction is consistent with the biomechanical demands of the two exercises, as reported by Barnes *et al*. (2019) [37], who found that squats impose greater peripheral fatigue than DLs due to higher quadriceps involvement. Together, these findings indicate that while both protocols allowed for recovery of gross CMJ performance by 24 h, the trajectory and magnitude of the initial impairment differed between exercises, with more immediate effects following BSQ and more subtle early changes after DL.

Moreover, reactive and temporal indices showed residual impairments, especially following the DL protocol. For example, RSImod remained reduced (Fig. 1f) and the durations of the eccentric phases were slightly prolonged, suggesting the persistence of neuromuscular fatigue that primarily affected reactive strength characteristics. The DL group also maintained slightly prolonged execution times (Fig. 1d), suggesting slower recovery of contraction velocity [63]. This pattern aligns with observations that increased contact duration after fatiguing protocols can act as a compensatory mechanism to generate impulse with temporarily weakened muscles [64]. In contrast, the BSQ group showed RSImod values that approached or slightly exceeded baseline at 24 h, likely due to re-acceleration of execution timing as peripheral fatigue subsided. Nonetheless, certain compensatory features persisted (e.g. elevated Yielding RFD or reduced Unloading RFD), suggesting that participants restored jump height through a less explosive and more temporally extended concentric strategy.

Taken together, these results indicate that although global CMJ performance may return to baseline within 24 h, phase-specific metrics reveal subtle residual deficits (particularly after DL exercise) that jump height alone may overlook. Even moderate reductions in RSImod indicate short-term impairments in reactive strength [49,63], underscoring the value of phase-specific CMJ analyses for identifying subtle forms of fatigue.

### Delayed muscle soreness and its relationship with neuromuscular fatigue

The DOMS results mirror the distinct fatigue-recovery profiles observed in CMJ-derived variables. Both BSQ and DL produced significant increases in soreness compared with control, peaking at 24 h and partially recovering at 48 h (Fig. 3). Although no statistical difference emerged between the two resistance exercises, the DL group showed slightly higher soreness at 24 h followed by a sharper decline, consistent with its delayed mechanical fatigue pattern seen in jump metrics. Conversely, the BSQ induced more immediate decrements in jump height, RSImod, and force variables, but with a faster recovery trajectory suggesting predominant neuromuscular fatigue rather than structural damage.

These findings align with prior evidence showing that knee-dominant exercises such as squats tend to place greater immediate demands on the quadriceps and reduce short-term power expression [37], whereas hip-dominant movements like DLs have been associated with higher eccentric loading of the posterior chain and with more delayed soreness responses [65,66]. However, the present study did not directly assess markers of muscle damage (e.g. creatine kinase), neuromuscular transmission, or central drive, and therefore any distinction between predominantly metabolic versus structural fatigue should be interpreted cautiously.

Based on the temporal patterns observed in CMJ-derived variables and DOMS, it is plausible that the two exercises elicited different recovery profiles, but the underlying mechanisms remain speculative. Rather than inferring specific physiological origins, our results support the broader conclusion that BSQs and DLs produce distinct time courses of neuromuscular impairment, which may warrant exercise-specific recovery strategies and the combined monitoring of both objective (CMJ) and subjective (DOMS) indicators.

### Limitations and future directions

This study also has some methodological limitations. First, although participants were instructed to perform all repetitions with maximal intended concentric velocity, we did not record actual mean velocity or velocity loss during the fatiguing sets. This prevents verification of the internal load and limits the precision with which fatigue intensity can be interpreted. Second, the use of different exercise modalities (the Smith machine for the BSQ and free weights for the DL) may have influenced both 1RM estimation and subsequent fatigue responses. This choice was made deliberately for reasons of safety and movement standardization, particularly during high-volume squatting, which carries a greater risk of technical breakdown. Nevertheless, it reduces ecological validity and limits comparability with free-weight squats. These factors should be considered when generalizing the present findings.

Moreover, future research should incorporate direct physiological and neuromuscular markers alongside CMJ outcomes. In particular, combining DOMS assessments with creatine kinase or other muscle-damage biomarkers would help clarify the extent to which BSQ and DL protocols differ in their peripheral muscular impact. Additionally, integrating surface electromyography (EMG) could improve understanding of muscle activation patterns and potential compensatory strategies.

Extending the monitoring period beyond 24–48 h (for example, to 72 h) would also provide a more complete characterization of recovery dynamics. Finally, studies involving elite or highly trained athletes may help determine whether greater training status attenuates neuromuscular decrements or modifies the balance between fatigue and potentiation.

## Conclusions

A single session of moderate-load, high-volume BSQ or DL exercise impairs explosive performance in the short term, although the recovery trajectory differs between exercises. The BSQ protocol used in this study produced an immediate and marked decline in countermovement jump performance, with jump height requiring ~24 h to return near baseline. In contrast, an equivalent DL protocol induced smaller acute reductions but resulted in residual impairments in neuromuscular speed and coordination after 24 h.

Selective reductions in RFD and lengthening of eccentric or concentric phases indicate that force-time curve-derived predictors are sensitive to specific fatigue components, even when jump height remains unchanged. Taken together, these findings suggest that phase-specific CMJ variables offer practitioners a more sensitive means of monitoring neuromuscular readiness and making informed decisions regarding recovery intervals and training load prescription, particularly in contexts involving moderate-load, high-volume resistance training.

## Supplementary Material

Table_S1_ldag013

## Data Availability

All raw and processed data underlying this study, including individual and aggregated biomechanical parameters from the countermovement jump analyses, are available from the corresponding author upon reasonable request.
